# Using Posterior EEG Theta Band to Assess the Effects of Architectural Designs on Landmark Recognition in an Urban Setting

**DOI:** 10.3389/fnhum.2020.584385

**Published:** 2020-12-11

**Authors:** James D. Rounds, Jesus Gabriel Cruz-Garza, Saleh Kalantari

**Affiliations:** ^1^Human Development, Cornell University, Ithaca, NY, United States; ^2^Department of Design and Environmental Analysis, Cornell University, Ithaca, NY, United States

**Keywords:** EEG, virtual reality, architectural design, landmark recognition, wayfinding

## Abstract

The process of urban landmark-based navigation has proven to be difficult to study in a rigorous fashion, primarily due to confounding variables and the problem of obtaining reliable data in real-world contexts. The development of high-resolution, immersive virtual reality technologies has opened exciting new possibilities for gathering data on human wayfinding that could not otherwise be readily obtained. We developed a research platform using a virtual environment and electroencephalography (EEG) to better understand the neural processes associated with landmark usage and recognition during urban navigation tasks. By adjusting the architectural parameters of different buildings in this virtual environment, we isolated and tested specific design features to determine whether or not they served as a target for landmarking. EEG theta band (4–7 Hz) event-related synchronization/desynchronization over posterior scalp areas was evaluated at the time when participants observed each target building along a predetermined self-paced route. A multi-level linear model was used to investigate the effects of salient architectural features on posterior scalp areas. Our results support the conclusion that highly salient architectural features—those that contrast sharply with the surrounding environment—are more likely to attract visual attention, remain in short-term memory, and activate brain regions associated with wayfinding compared with non-salient buildings. After establishing this main aggregate effect, we evaluated specific salient architectural features and neural correlates of navigation processing. The buildings that most strongly associated extended gaze time, location recall accuracy, and changes in theta-band neural patterns with landmarking in our study were those that incorporated rotational twist designs and natural elements such as trees and gardens. Other building features, such as unusual façade patterns or building heights, were to a lesser extent also associated with landmarking.

## Introduction and Background

More than 55% of the world's population currently resides in urban environments, and that percentage is expected to increase in upcoming decades (United Nations, 2018). The architectural design of these built environments can have a significant impact on those who live there (Kalantari and Shepley, 2020). Numerous cognitive and health-related effects associated with urban design have been documented in recent years (e.g., Nasar, 1994; Heft and Nasar, 2000; Knöll et al., 2018; Kondo et al., 2018). However, in some topical areas it has been difficult to obtain rigorous empirical data about the effects of urban design. Wayfinding is one of these problematic areas. There has long been speculation and anecdotal evidence that the design of cities can make wayfinding much easier or harder for people who live in and visit these environments—Tolman (1948) introduced the concept of “cognitive maps” to discuss neural wayfinding processes, a concept that was later used by Kevin Lynch, in *The Image of the City* (1960), to explain human wayfinding in the urban environment. More recently, researchers have adjusted and refined this analysis to discuss the specific mental processes that are involved in learning routes and establishing orientation in cities (Blades et al., 2002; Julian and Epstein, 2013), as well as the ways in which people make use of navigation tools such as signs and maps (He et al., 2015). Gradually, this research is beginning to take on a more rigorous character, with a growing interest in collecting both behavioral and neurological data.

One crucial aspect of wayfinding that has been gaining increasing scholarly attention is the use of landmarks. May et al. (2003) found that landmarks are the most common type of cue used for navigation by pedestrians in urban centers. Lee et al. (2006) demonstrated that the availability of landmarks greatly reduces the number of mistakes made during wayfinding, and that landmarks also reduce the stress of wayfinding decisions. Specific studies have identified landmark-use in a wide array of navigation contexts, including city exteriors and the internal navigation of hospitals, airports, train stations, and other large urban structures (Fewings, 2001; Joseph, 2006; Epstein and Vass, 2014; Chang and Zheng, 2016; Sharma et al., 2017). Several studies have found that landmarks facilitate the development of spatial orientation skills in children (Howard and Templeton, 1966; Acredolo, 1977; Sadalla et al., 1980) and that they are particularly important wayfinding tools for older adults (Burns, 1997; Bradley and Dunlop, 2005). Other researchers have noted that landmarks are frequently used when one person attempts to communicate a route, covey a mental model of an environment, or give directions to another individual (Siegel and White, 1975; Egenhofer and Mark, 1995; Maass and Schmauks, 1998; Lovelace et al., 1999; Michon and Denis, 2001; Tenbrink, 2008).

*Saliency*, which refers to the contrast between a landmark and its surrounding terrain, is widely recognized as a central component of landmark selection (Clark, 1996; Sorrows and Hirtle, 1999; Raubal and Winter, 2002; Caduff and Timpf, 2008). Landmarks generally need to stand out from their surroundings in order to be easily recognized and used as spatial reference points in memory and in communication. If individuals are required to use landmarks with low saliency, then the wayfinding process will be more difficult and time-consuming, with commensurate increases in cognitive burdens and a greater likelihood of wayfinding errors (Sorrows and Hirtle, 1999). Various kinds of landmark saliency have been proposed by researchers, including distinctive visual patterns or colors, structural/geometric anomalies, and memorable cultural associations (Sorrows and Hirtle, 1999; Raubal and Winter, 2002; Caduff and Timpf, 2008; Grabler et al., 2008). In the design field, there have been numerous attempts to quantify and categorize the specific saliency factors that can promote landmark identification and recognition, with the goal of improving the design of built environments and making wayfinding easier (Raubal and Winter, 2002; Nothegger et al., 2004; Klippel and Winter, 2005; Aziz and Mertsching, 2007; Claramunt and Winter, 2007; Duckham et al., 2010; Zhang et al., 2014). Some researchers have even developed algorithmic and data-mining methods to automatically identify potential salient landmarks in virtual spaces or in architectural design plans (Elias, 2003; Peters et al., 2010; Winter et al., 2008).

Despite all of this research on landmark salience, very little scholarly consensus has emerged regarding what features in architectural design are most likely to produce readily identifiable landmarks for use by diverse human populations. Part of the reason for the conflicting and inconclusive nature of these studies may be methodological. Most of the previous studies on urban landmarking have been based on showing participants pictures of urban scenes and asking them to find or identify landmarks. This approach is limited due to the static, two-dimensional impression given by the pictures, which may not reflect the same saliency factors that are present when people move through complex, dynamic, and immersive environments.

Another concern in the existing landmarking design literature is that these analyses have relied mostly on behavioral and/or self-reported data (Cornwell et al., 2008; Pu et al., 2017; Sharma et al., 2017). Less attention has been given to the possibility of collecting neurological data (such as EEG signals) as a more robust scientific basis to measure covert processes and to distinguish cause and effect in human responses to design features. EEG methods are a promising tool to characterize the interplay of neural states and information processing (Banaschewski and Brandeis, 2007). The goal of the current study was to address these concerns by developing an assessment method for landmark recognition using spectral analyses of scalp EEG electrodes over neural regions that have been implicated in spatial awareness and spatial memory. The use of an immersive, three-dimensional virtual environment (as opposed to static pictures) allowed the researchers to differentiate experimental conditions by making targeted adjustments to specific landmark design variables. It also enabled the collection of cleaner real-time neural data while participants completed active navigational and recall tasks (compared to real-world navigation that would generate motion artifacts in the EEG).

### Brain Activity Related to Landmark Recognition

In an influential study, Epstein and Vass (2014) identified four cognitive mechanisms related to landmark- based navigation. These include: (a) the landmark-recognition mechanism, which clarifies “what am I looking at?”; (b) the localization/orientation process, which clarifies “where am I and which direction am I pointing?”; (c) the encoding and retrieval system for spatial knowledge, which clarifies “where are other specific places in relation to my position?”; and (d) the route-planning process, which solves the problem of “how do I get from here to another specific place?” In the present study, participants were tasked with passively observing buildings along a simulated urban route, and then asked to later recall where several “target” buildings were along the route. Therefore, of these mechanisms we hypothesized that more salient building features would increase activation of brain regions and networks associated with “(b)”, localization/reorientation, and “(c)”, encoding and retrieval of spatial knowledge.

Recent EEG and fMRI work has demonstrated that the task demands of integrating allocentric and egocentric information during spatial navigation are strongly associated with activity in the retrosplenial cortex (RSC; Auger et al., 2012; Lin et al., 2015; Fischer et al., 2020), as part of network of spatial awareness and memory centers including the hippocampus [HC], occipital place area [OPA], and the parahippocampal cortex [PHC], which includes the parahippocampal place area [PPA]; (Wolbers and Büchel, 2005; Hanslmayr et al., 2011; Julian et al., 2018). The RSC is generally located below the inferior and superior posterior scalp areas (e.g., EEG sites P3, P1, CP3, CP1 on the left, and P2, P4, CP2, CP4 on the right), and is highly amenable to EEG measurement, although its activity can be hard to disentangle from that of the PHC and posterior parietal cortex in general. And certainly, EEG data is limited in its spatial resolution, for several reasons. But carefully designed experiments with rodents and humans have revealed that the RSC seems uniquely involved in integrating “egocentric” spatial awareness—the participant's sense of their current spatial position and direction in relation to previous personal positions and actions—with incoming “allocentric” information about the relative position of external objects in the environment during movement (Buzsáki, 2005; Dhindsa et al., 2014; Lin et al., 2015).

Multimodal evidence supports the role of theta band EEG, and the connectivity between the RSC and other areas, in this path integration process. In a tractography-based study, Ramanoël et al. (2019) found correlations between spatial memory skills and age-associated deficits in resting state structural connectivity between the left RSC and Hippocampus. And in a recent study involving patients with mild cognitive impairment and prodromal Alzheimer's, virtual navigation skills were significantly negatively correlated with levels of disease biomarkers in RSC and HC (Howett et al., 2019). Support for the mechanistic role of theta activity among these regions comes from simultaneous EEG-fMRI experiments that show a negative correlation between Default-Mode Network activation (turning attention inward) and theta power in the superior posterior parietal and frontal regions (Scheeringa et al., 2009; however, see Zumer et al., 2014), and that increased theta power during encoding (across several regions anti-correlated with the default mode network) predicts which encoded information is later remembered (White et al., 2013).

It follows naturally that researchers would look to the RSC and related areas when measuring the effect of different navigation strategies, as well as the effect of different environment features. Auger et al. (2012) found that RSC activity while viewing landmarks correlated with an individual's skills as a navigator, and specifically when viewing buildings deemed more “permanent,” suggesting this brain area's role is to help tag especially meaningful spatial cues during memory formation. And Lin et al. (2015) found that a participant's natural navigation strategy preference (egocentric vs. allocentric) in a virtual navigation task was associated with several EEG markers, such as theta and alpha synchronization and desynchronization in sources localized to the retrosplenial and posterior parietal cortices during turning and new-scene encoding (among other findings). Previous research has also shown a scalp-wide increase in theta power after making a turn decision in a virtual navigation task (Bischof and Boulanger, 2003), and during decision-making phases in navigational tasks (Jaiswal et al., 2010). The theta band signals are thought to act as a mechanism by which different neuronal groups and regions synchronize with each other in order to accomplish objectives (Buzsáki, 2005). In both human and animal studies, theta activation in posterior parietal regions has been observed to feature prominently in navigational tasks (White et al., 2011; Belchior et al., 2014) and goal- directed environmental information processing (Cornwell et al., 2008; Pu et al., 2017), as well as in memory formation and recall (Paller and Wagner, 2002; Jaiswal et al., 2010; Vaidya and Johnston, 2013; Koike et al., 2017; Scholz et al., 2017). Based on these prior findings, in our study we focused on measuring theta activity in the medial and lateral superior posterior scalp locations while participants gazed at the target buildings of different hypothesized saliency levels.

We measured theta activation by calculating event-related desynchronization/ synchronization (ERD/ERS) averaged across the 4–7 Hz EEG frequency band, during periods (minimum 1.5 s) when participants gazed at the target buildings. A 1 min resting period was used as a baseline for comparing task-related theta-power changes, following Pfurtscheller and Lopes da Silva (1999), and Sharma et al. (2017).

### The Use of Virtual Reality in Navigational Behavior Research

Today's high-resolution virtual environments are becoming astonishingly lifelike, opening new opportunities to study various types of human behavior in a controlled context. The use of virtual reality (VR) is already widespread in behavioral (Makransky et al., 2019), cognitive (Wolbers and Büchel, 2005), medical (Plancher et al., 2012; Clay et al., 2020), and design research (Kalantari and Neo, 2020). This technology allows researchers to isolate and adjust environmental variables in a way that would not be possible in the real world. For example, researchers can easily add or remove windows from an otherwise identical building, or change the color or pattern of a building's façade, without incurring any construction expenses. These types of design studies have the potential to provide an enormous wealth of empirical data (Jeffery, 2019). We must always remain aware that there are possible discrepancies between virtual and real-world results, but research using virtual environments is in many ways superior to prior studies that relied on static images and/or were rife with confounding variables. In many cases virtual platforms can provide an important way to obtain feedback about design questions that otherwise could not be rigorously tested at all.

With the groundwork for the VR largely in place, the use of this technology as a design research tool mostly requires the technological know-how to make targeted modifications in the virtual environment— along with any coding that is necessary to implement other desired research features, such as presenting real- time interactive questionnaires to study participants within the environment. Previous researchers have demonstrated important concordances between human behavior in real-world navigation and virtual navigation (Werner and Schindler, 2004; Jansen-Osmann et al., 2007; Jiang and Li, 2009; Tang et al., 2009; Kuliga et al., 2015; Slone et al., 2015; De Tommaso et al., 2016). Many of these studies used virtual contexts to develop hypotheses about behavior that were later confirmed in real-world environments. Of particular note for the current research are studies by Gazova et al. (2013) and Nys et al. (2015), which used VR as a research tool for evaluating the relative importance of landmark use during wayfinding. Similarly, Röser et al. (2012) used a virtual environment to evaluate the ideal position of a landmark at an intersection.

In the current study participants were immersed in a high-resolution VR urban environment and asked to complete various navigational tasks. In contrast with physical environment navigation, this allowed us to use neurophysiological sensors to better understand the impact of different architectural landmark designs during navigational memory formation. The use of the virtual environment made it possible to collect robust neurological data while reducing motion or sweat-related artifacts in the EEG signals. In addition, it allowed the researchers to carefully and precisely create the desired urban design testing environment and to readily move or substitute individual buildings and features during preparation, or condition creation, thus helping to isolate specific design variables. Our primary focus was to triangulate behavioral and neurological responses to different architectural designs during navigation and to determine if certain aspects of these designs may assist or hinder in the identification of landmarks for navigation and recall.

#### Hypotheses

Our broadest hypothesis was that buildings useful as landmarks would have a relatively high “saliency” factor, which is defined in navigational studies as a striking feature that stands out from the surrounding information terrain (Sorrows and Hirtle, 1999; Raubal and Winter, 2002; Caduff and Timpf, 2008). The term “saliency” has several related, yet distinct, meanings. In perception and cognition studies, for example, a red flower against a background of green foliage has a high perceptual saliency, as does a loud noise in a quiet room, or an object in motion in an otherwise still environment (Koch and Ullman, 1985; Kerzel et al., 2011). It is important to note that the landmark saliency of architectural design is relative to the surrounding urban environment. In a city where nearly all of the buildings were geodesic domes, for example, an ordinary square apartment building might stand out as a striking landmark. If an object in a scene were also especially novel, the initial effect would be similar. For the purposes of this study we used currently existing Western metropolitan architecture as the urban background, and categorized buildings as salient or non-salient based on variations from this environmental norm along several different design features: relative height, footprint-shape, twist, and façade design. We aimed to control for the previously discussed overlapping influences of saliency and novelty during the creation of the virtual environment by: (a) ensuring that all the target buildings were similar to each other regarding how distinct they were from the surrounding background in major low-level features, e.g. luminance, color, general style; and (b) making sure that all the target buildings were generally plausible, and not exceptionally unusual.

Previous studies have noted in an anecdotal fashion that striking architectural design features in a building can be related to people's tendency to regard that building as a landmark (Lynch, 1960; Darken and Peterson, 2001). We expected that the concept of environmental/landmark saliency can help to explain this correlation between specific design features and the prominence of a building in human visual memory. During viewing of these more salient buildings, we would therefore expect to see increased posterior superior scalp EEG theta power, (i.e., synchronization) as one indicator of increased processing in response to relevant navigational cues. To support this overall perspective, we tested the following specific hypotheses:

Hypothesis H: Mean recall accuracy, user interaction (gaze duration), and neural signatures of spatial awareness (superior posterior theta power), will be heightened when participants gaze at salient buildings as compared to non-salient buildings.

- Hypothesis H1: Recall accuracy, user interaction (gaze duration), and neural signatures of spatial awareness (superior posterior theta power) will be heightened as a linear function of how salient a viewed building is, e.g. strongly salient, weakly salient, weakly non-salient, or strongly non-salient.

Upon testing for this aggregate main effect (Hypothesis H), as well as dose-response model of increasing salience (H1), we then drilled down to see if the same outcome measures tested above will be heightened in response to viewing buildings with specific salient architectural features compared with buildings that lack those specific architectural features.

- Hypothesis H2: Recall accuracy, user interaction (gaze duration), and neural signatures of spatial awareness (superior posterior theta power) will be heightened when participants gaze at buildings that are *taller than surrounding buildings*.
- Hypothesis H3: Recall accuracy, user interaction (gaze duration), and neural signatures of spatial awareness (superior posterior theta power) will be heightened when participants gaze at buildings containing *biologically inspired and natural elements*.
- Hypothesis H4: Recall accuracy, user interaction (gaze duration), and neural signatures of spatial awareness (superior posterior theta power) will be heightened when participants gaze at buildings containing a *twisted architectural façade or unusual footprints*.

## Materials and Methods

Drawing from the previous theoretical literature on landmark identification (Raubal and Winter, 2002; Klippel and Winter, 2005; Duckham et al., 2010; Peters et al., 2010), and focusing on the concept of visual saliency, we designed twelve different buildings to be tested in the virtual urban environment. These buildings were created using a parametric modeling algorithm in the Grasshopper software platform (www.grasshopper3d.com) so that their design features could be readily adjusted. The overall building contours were defined by their height, footprint-shape, and various aspects of their façade design. Color saliency was used as a way to make all of these buildings stand out to some extent from their background (Aziz and Mertsching, 2007). The buildings under investigation were presented using white colors, while all other background buildings were presented in gray and opaque colors. The background buildings were also designed to unobtrusively mirror typical Western metropolitan environments, thereby helping to reduce their structural saliency (Raubal and Winter, 2002). Designing the target buildings like this ensured that they shared numerous low-level perceptual features with each other, regardless of their level of architectural “salience,” especially in contrast with the background urban scenery and architecture. This helped ensure that none of the target buildings would attract attention due to the “novelty” of low-level features, more so than the others. One important distinction to make is between saliency and novelty. In one sense, this can mean that new items in an environment can gain saliency over familiar items simply as a result of being new, somewhat regardless of their other features (Stirk and Underwood, 2007). In another sense, it can mean that an object with a very unique feature, e.g., a shape or pattern, that a person has never experienced before may make it highly salient to that person, even if the object's other low-level features are not distinctive or salient in the traditional sense (Underwood et al., 2009). We attempted to control for novelty while manipulating saliency.

For the height variable, we were primarily interested in testing relative height compared to the environmental background, so we also included a contextual height difference variable, which indicates whether a building is taller or the same height compared to surrounding structures. For the footprint variable we used a square, a triangle, a pentagon, and a circle. We included a “twist” variable in some designs, meaning that the building outline had a twisted form in the upward direction (z axis). Some of the buildings included a horizontal overhang. The study incorporated five different façade patterns, including a design with dominant horizontal lines, one with strong vertical lines, one with a grid pattern, one with a biologically inspired abstract Voronoi pattern, and one that included natural elements such as integrated gardens. Based on the overall prior evidence about the effects of building size, shape deviation, and façade eccentricity on visual saliency (Itti and Koch, 2000, 2001; Raubal and Winter, 2002; Zhang et al., 2014) we intentionally created seven buildings with salient features in height, shape, or façade patterns, and created five non-salient buildings to evaluate the psychophysiological responses across these two categories (Table 1).

**Table 1:** Landmark buildings included in the experiment were coded and described according to a basic set of design variables including height, footprint, and façade pattern.

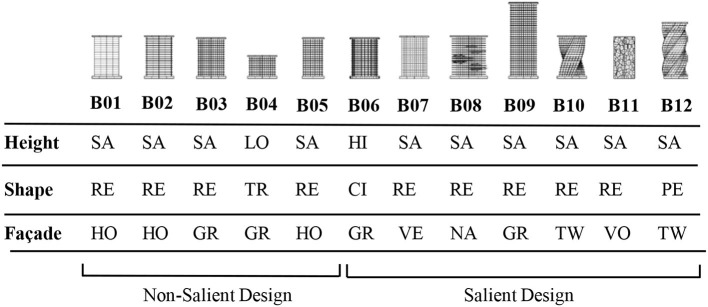

*SA, Same level with neighbor buildings; LO, Lower than neighbor buildings; HI, Higher than neighbor buildings; RE, Rectangular; TR, Triangle; CI, Circle; PE, Pentagon; HO, Horizontal lines pattern; GR, Grid lines pattern; VE, Vertical lines pattern; NA, Nature feature included in the façade; TW, Twisted façade type; VO, Voronoi pattern*.

### Virtual Reality Development

The creation of the virtual urban environment was carried out using Epic Games' Unreal Engine (www.epicgames.com). Most of the modeling and UV-mapping took place within Autodesk Maya. Parametric modeling for building exteriors was performed in Maya using Python and Mel scripting. Texturing was done procedurally using the Substance software platform, at a resolution of 4096 × 4096. The Unreal Engine uses blueprint scripting, which can allow for a quick learning curve on the part of researchers and designers who may want to expand or replicate our work. All of the front-end interaction and user interactivity in our environment also leverages the blueprint platform.

**Figure 1:**
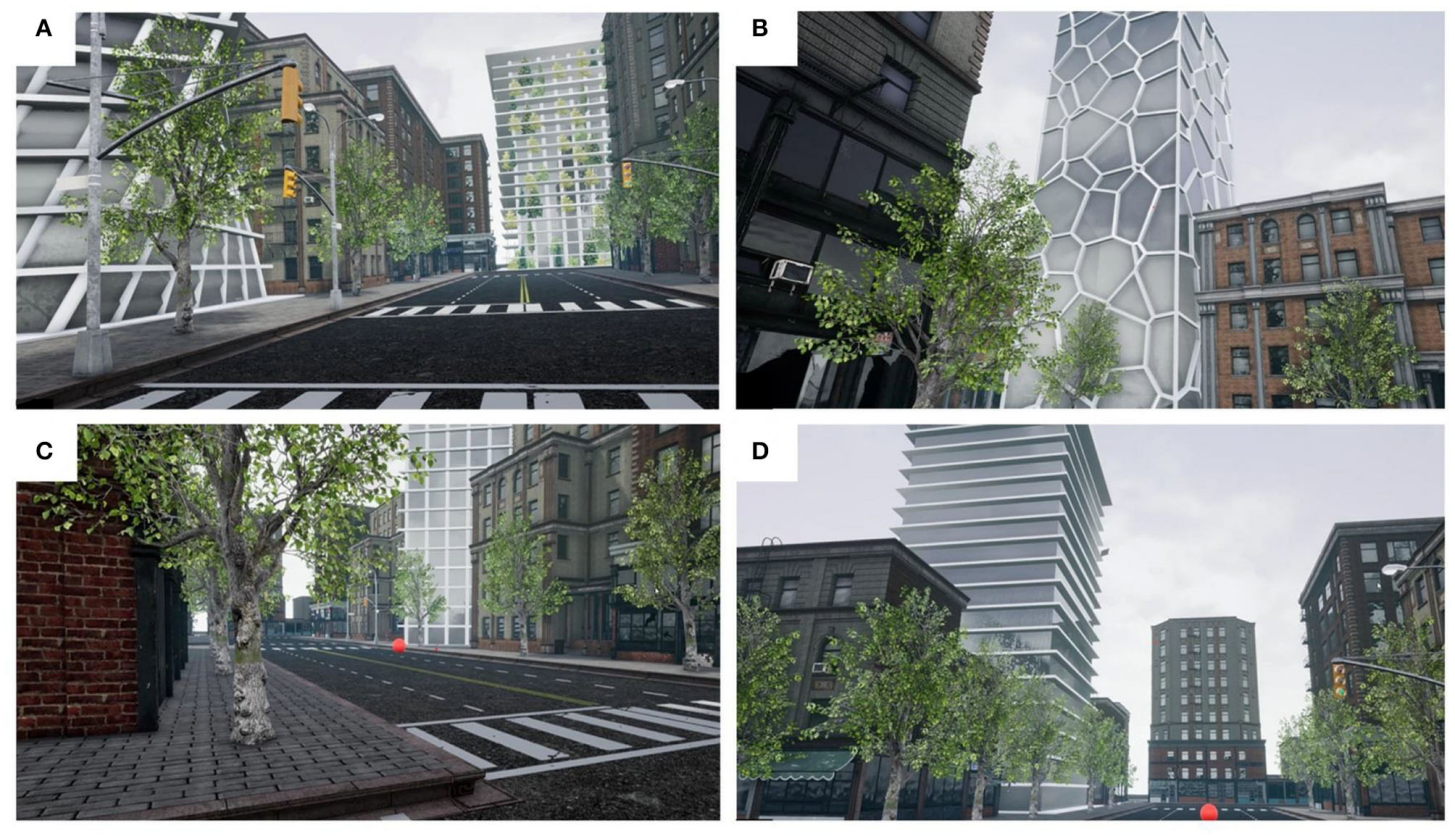
Immersive virtual environment of an urban setting; screenshots from a participants' perspective. **(A)** Square footprint, twist-façade building to the left, with the Nature-façade building at the end of the street segment. **(B)** Voronoi-façade building. **(C)** Square-footprint Grid-façade building. **(D)** Square-footprint Horizontal-façade building. The red sphere indicates the next location to travel.

The test buildings were integrated into an interactive and user-friendly VR environment simulating a standard North American urban exterior. We set the camera position at 1.70 m above the street (corresponding to average human eye-height). The environment was presented to study participants using an Oculus Rift head-mounted display (HMD) through a gaming laptop with a resolution of 1,920 × 1,200 pixels. The Oculus HMD provides a 100-degree horizontal field view with 75 Hz refresh rate, and can be adjusted for participants with different interpupillary distances. Some examples of two-dimensional screen captures from the virtual environment are shown in Figures 1A–D.

### Participants

Twenty-nine healthy human adults with normal or corrected-to-normal vision were recruited for the study by word of mouth and broadly distributed email announcements within various departments of the academic institution. After exclusion, (see “Data Exclusion” below,” data from twenty-one participants were analyzed. The participants' ages ranged from 18 to 55 years (*M* = 27.65, *SD* = 10.04). The majority of the participants were university students (*n* = 19), and the rest were academic faculty members. All of the participants were associated with the University of Houston, representing the departments in Engineering, Biological Sciences, Humanities, Economics, and Computer Sciences. All participants gave informed written consent before participating in the experiment and were compensated with a $25 gift card at the end of the study. The participants represented various national backgrounds (U.S., India, and Mexico were most common), and had a variety of ethnic backgrounds: nine reported as Asian, seven as Latino and/or Hispanic, one as Middle Eastern, and four as White (Non-Hispanic). Eight of the participants reported as women, thirteen as men, and none as other. The participants were also asked to report their sleep patterns and rate their mental fatigue level (M = 3.95, on scale from 1, low fatigue to 10, high fatigue) at the beginning of the experiment. Thirty-three percent reported getting seven or more hours of sleep the previous night, 29% reported between 6 and 7 h of sleep, and 38% reported 4 to 6 h of sleep. Participants were free of current neurological conditions, abstained from any psychoactive substances prior to the study (except for two who had recently consumed caffeine, within 1 h), and only two participants were actively taking psychological medication. Participants were asked in a post-experiment survey to indicate their level of stress or discomfort with the virtual reality experience and the EEG headset, (1=comfortable with no stress; 10 = uncomfortable and stressful), and to report how realistic the virtual environment seemed (1=not similar; 10=very similar). Participants indicated having no familiarity with virtual reality technology, and they reported that the VR system (*M* = 4.083, *SD* = 2.465) and EEG headset (*M* = 3.75; *SD* = 2.156) were not uncomfortable or stressful. Participants found the VR environment realistic (*M* = 6.33; *SD* = 1.66).

### Procedure and Tasks

The experiments were conducted in a laboratory setting at the University of Houston. The study protocol was approved by the University of Houston's Institutional Review Board to protect the privacy and safety of the participants. After filling out the consent form and demographic forms, participants were fitted with the biometric sensors (described in more detail below). The initial stages of the experiment took place without the use of a VR headset. To establish baseline biometric data, the participants were asked to sit quietly facing a blank computer monitor for 1 min. Once this baseline data was collected, the participants donned the VR headset and entered the virtual environment. They were given an initial 5-min period in the VR to become accustomed with the navigation tools and to explore the platform.

The participants were then asked to travel along two different routes through the virtual city (with a brief break in between), and both routes included the twelve “target” buildings but in different locations. Participants “moved” through the virtual environment by using a Microsoft Xbox controller button-press to advance at their own pace along pre-specified locations on the path. Immediately after completing each route, the participants were asked to identify the general region/location of five different target buildings (as a result, there were a total of 10 items in the memory recall assessment). At the end of the experiment, the participants filled out an exit survey to provide additional feedback about their reactions to the study setting, the virtual environment, and the experimental design.

### Signal-Derived Metrics

**Figure 2:**
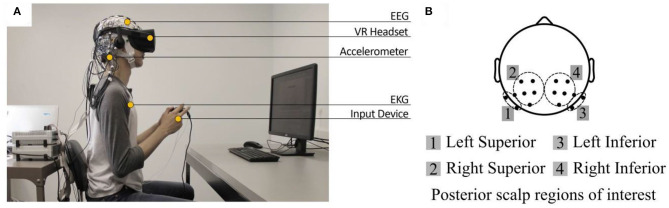
Experimental setup. **(A)** Measurement equipment and experimental interface. The experiment participant navigated through the virtual environment using a hand-held controller. **(B)** Posterior scalp regions of interest for EEG analysis.

To obtain measurements of their biometric responses, the participants were instrumented with a non-invasive electroencephalography (EEG) cap to record electrical activity in their brains, and appropriately-placed sensors to record eye muscle movements (electro-oculography, EOG), heart activity (electrocardiogram, ECG, not analyzed for this experiment), skin conductance (galvanic sensor response, GSR, not analyzed for this experiment), and head motion (tri-axial head accelerometer, not analyzed for this experiment) (Figure 2A). All of this data was recorded at 500 Hz and synchronized using the 64-channel ActiCHamp module (Brain Products GmbH, Germany) with Ag/AgCl active electrodes. A total of 63 electrodes were used (57 for EEG, 4 for EOG, and 2 for ECG). Only EEG data was analyzed in this study, although EOG data was included during ICA to help with IC-based artifact elimination. We focused our data analysis on the Left Posterior Superior scalp region (electrodes CP3, CP1, P1, P3, P5, and PO3); the Right Posterior Superior scalp region (electrodes CP2, CP4, P6, P4, P2, and PO4); the Left Parietal Inferior region (electrodes P8 and PO8); and the Right Posterior Inferior scalp region (electrodes P7 and PO7). These regions are shown in Figure 2B. The two Posterior Inferior regions are displayed as slightly off the scalp since in their true position they are not easily viewed from a top-down viewpoint. Lab-Streaming Layer (LSL), a multi-modal data collection software, was used to synchronize all modes of data (Kothe, 2014).

### EEG Data Processing

The EEG data were analyzed using the EEGLAB software package (Delorme and Makeig, 2004). Raw “xdf” data files were imported at their original sampling rate of 500 Hz, low-pass filtered at 100 Hz, high-pass filtered at 0.1 Hz, and then run through the “Cleanline” algorithm, which selectively filters out the 60 Hz power-line noise using an adaptive frequency-domain (multi-taper) regression technique. The chronological segment of the EEG data relevant to the experimental procedure was then extracted and run through the PREP Pipeline (Bigdely-Shamlo et al., 2015), which is a robust re-referencing method that minimizes the bias introduced by referencing using noisy channels. Artifact-laden time-windows and channels were identified using manual inspection and deleted, by research team members blind to the order of the trial conditions. These trimmed datasets were then each run through Independent Component Analysis (ICA), using the extended Infomax variation as implemented in the EEGLAB package. ICA is a form of second- order blind identification, which performs spatial unmixing to identify underlying sources of signal within an under-specified space (Onton et al., 2006), such as EEG. Independent Components indicative of eye- movements and muscle activity were identified and deleted, as well as time windows with gross artifact contamination.

### Data Exclusion

Several participants' data were excluded from final analyses. Final EEG analyses included 21 participants: two were excluded due to extremely high theta power activity (> 3 St.Dev. of the overall mean theta Event-Related Desynchronization); three were excluded due to low EEG recording quality (> 33% of channels exceeding 100 μV for >50% of time series); and three were excluded because they did not gaze at a sufficient number of target buildings for longer than 1.5 s each (1.5 s was the *a priori* duration threshold used to include time-points for analysis).

### Data Analysis

#### Gaze Time

We used recorded screen-capture video from the experiment to identify the periods of time when the participants observed the buildings that were being tested. The start and end of these time periods were determined based on the content of the VR display, which itself was linked to the direction of the participants' gaze. One researcher marked all of the screen data using video-editing software (Camtasia), and then another researcher reviewed the marked segments for accuracy. These gaze event markers were exported for analysis, and to be automatically imported into the EEG files as events. We analyzed the total amount of time that participants spent looking at each building (gaze time), as well as the number of instances in which their gaze returned to the building (gaze count). These measurements of gaze times and counts were averaged across both of the routes that were tested (route 1 and route 2) (Supplementary Table 1).

We used linear models in R [using the lm() function] to determine if participants looked for longer periods of time at certain types of buildings, with Overall Building Saliency and various building design features as the predictor variables in separate tests for each hypothesis, and average Gaze Time as the outcome variable. Paired *t*-test comparisons were then computed, using a tukey method for *p*-value adjustments. Gaze times of 0 s were excluded from the statistical analyses (< 1% of all cases).

#### Location Recall Accuracy

After completing each route, participants were presented with a series of images of target buildings, alongside a map of the route that they had just traversed. The map was divided into four zones (A, B, C, and D).

Participants were asked to identify the zone in which each test building appeared. Only ten of the buildings were tested for visual memory (omitting the B01and the B09 buildings). In the analysis of the visual memory test data, we used linear models to determine if participants more accurately remembered the zone locations of certain types of buildings. Overall Building Saliency and various building design features were used as the predictor variables, and accuracy of visual memory was used as the outcome variable.

#### EEG Theta Power ERD/ERS as Participants Observed the Buildings

The mean Event-Related Synchronization/Desynchronization (ERD/ERS) was calculated across all of the EEG electrodes in each scalp region, averaged across the theta-band frequencies (4–7 Hz), for the combined duration of the time periods during which a participant was viewing a particular test building. Welch's method of overlapped segment averaging was used as an estimator of power spectral density, as implemented in EEGLAB's “spectopo” function. EEG power spectral density values were converted to μV^2^/Hz units, so that all theta power values would be positive. The ERD/ERS value was then calculated as:

$$\begin{array}{l} \%\text{ ERD}/\text{ERS}=\frac{A-R}{R}\;\times \;100 \end{array}$$

where *R* indicates baseline reference data and *A* indicates the theta power value associated with the time in which a participant viewed a particular test building (Pfurtscheller and Lopes da Silva, 1999). Since the viewing time differed for each participant and for each building, the amount of data that was fed into each ERD calculation varied. These ERD values were entered into a separate mixed multilevel model for each planned comparison, with scalp region of interest and building design features as fixed effects, participant ID as a random effect, and mean theta ERD as the dependent outcome variable. Paired *t*-test comparisons were then computed, using a Tukey method for *p*-value adjustments.

## Results

Each hypothesis was tested using a separate linear model, using the building categories relevant to the variables being evaluated: Non-salient vs. Salient buildings, relative height, and façade designs (Nature vs. non-nature, and Twist vs. non-twist).

### Gaze Time Looking at Buildings

A high building Saliency as defined by Table 1 was associated with longer Gaze Time in the two- category (i.e. Salient and Non-salient) comparison (*F*_(1, 390)_ = 27.60, *p* < 0.001). We also found associations between several architectural features and the average Gaze Time. The Vertical and Voronoi façade patterns, in particular, attracted significantly longer Gaze Times compared to other façade types (all |t|s > |−2.84|, ps < 0.03). We observed longer Gaze Times for the buildings with the salient feature of Twist façade design, compared with non-twist façade building designs (*F*_(__1, 54)_ = 26.74, *p* < 0.001). Longer Gaze Times were found for the Nature façade design compared with non-Nature designs (*F*_(__1, 54)_ = 14.67, *p* < 0.001); and longer Gaze Times for contextually the taller building compared to those with the same height level with the neighbor buildings (*F*_(__2, 109)_ = 6.74, *p* < 0.001).

### Location Recall Accuracy

**Figure 3:**
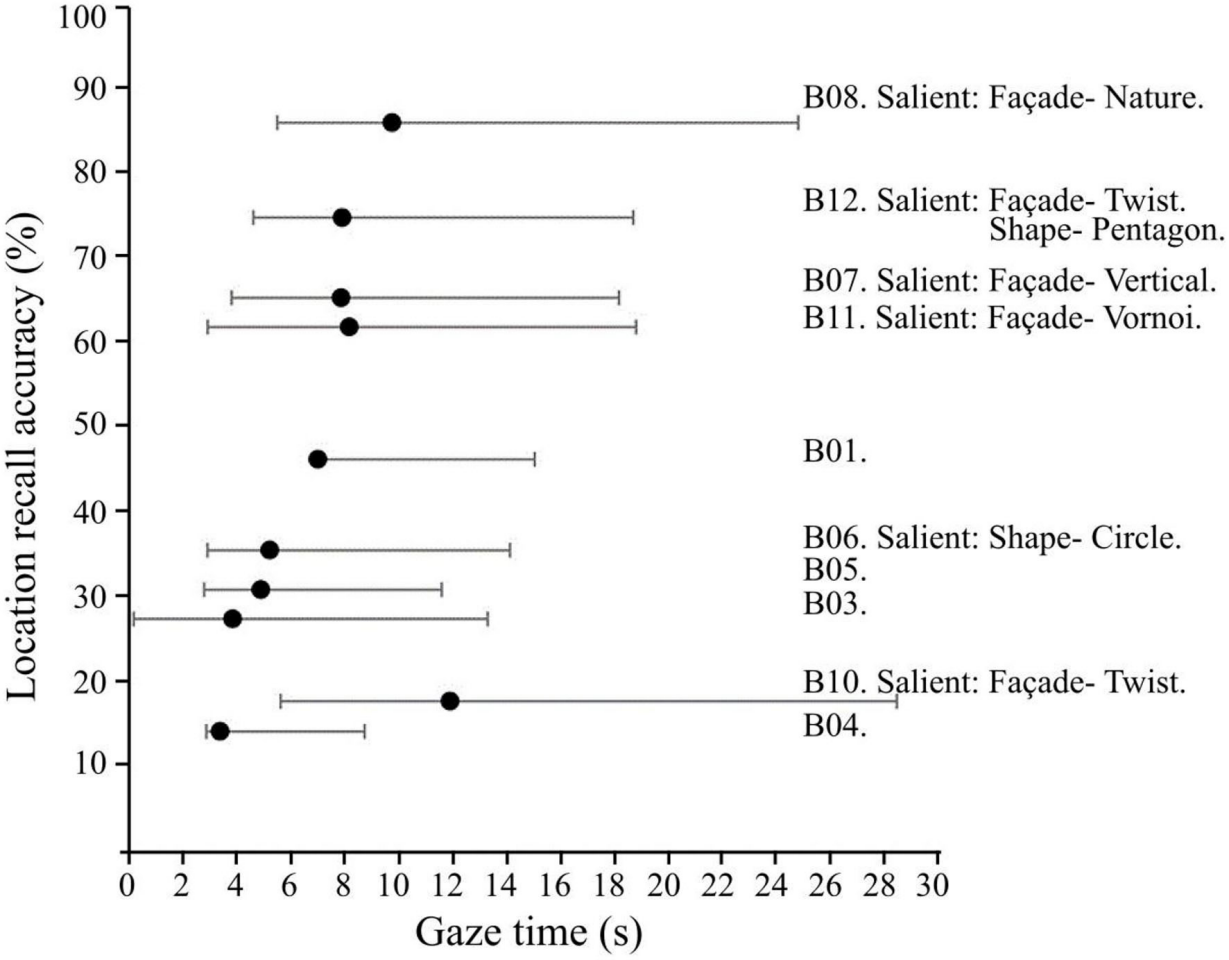
Location recall accuracy compared with gaze time distributions, with error bars showing the 25th and 75th percentile.

Building Saliency was associated with more accurate visual memory in the two-category comparison (*F*_(__1, 283)_ = 22.26, *p* < 0.001) of Non-Salient vs. Salient buildings (Figure 3). Similar to the gaze-tracking results, more accurate visual memory was associated with the Vertical and Voronoi façade patterns (both ts > 5.2, *p* < 0.001), and with the Nature façade design (*F*_(__1, 56)_ = 30.19, *p* < 0.001), compared with their respective matched non-salient buildings. In contrast to the gaze-time results, no significant overall association was found between visual memory accuracy and the Twist façade design feature. While the building B12 (Pentagon-footprint, Twist-façade design) obtained high location recall accuracy in the visual memory tests, building B10 (Square footprint, Twist-façade design) obtained low location recall accuracy. No contextually tall buildings were tested for visual memory, so we lack the data to evaluate the association between visual memory accuracy and contextual height. Buildings B02 and B09 were not included in the recall task, simply as part of the effort to keep the experiment duration as short as possible.

### EEG Theta Power ERD/ERS as Participants Observed the Buildings

**Figure 4:**
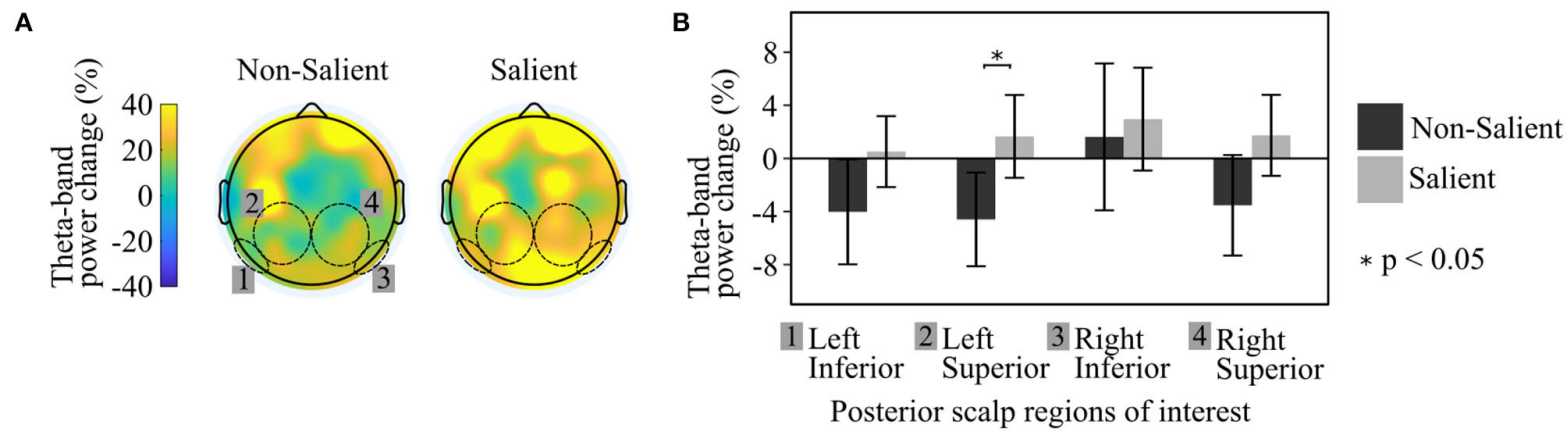
Grand Average Scalp Maps of theta (4–7 Hz) ERD/ERS when participants observed the buildings inside the VR environment. Comparison of Salient vs. non-Salient buildings. **(A)** Scalp map theta power change compared to baseline. **(B)** Statistical comparison in the four posterior scalp regions of interest.

The multilevel linear model for building Saliency indicated a significant association with theta activation (*F*_(__1, 140)_ = 8.343, *p* = 0.005), i.e., Event-related synchronization (ERS), thus supporting the main effect hypothesis H (Figure 4). Individual *t*-tests comparing these conditions within each of the four scalp regions of interest revealed that theta power changes over baseline, for more salient buildings, were significantly greater in Left Posterior Superior region (*t* = −2.08, *p* = 0.04), and were trending significantly for the Right Posterior Superior region (*t* = −1.75, *p* = 0.08) (Figure 4). As a result of these findings, our hypotheses were tested specifically over theta power changes (ERD/ERS) in the Left Posterior Superior region.

**Figure 5:**
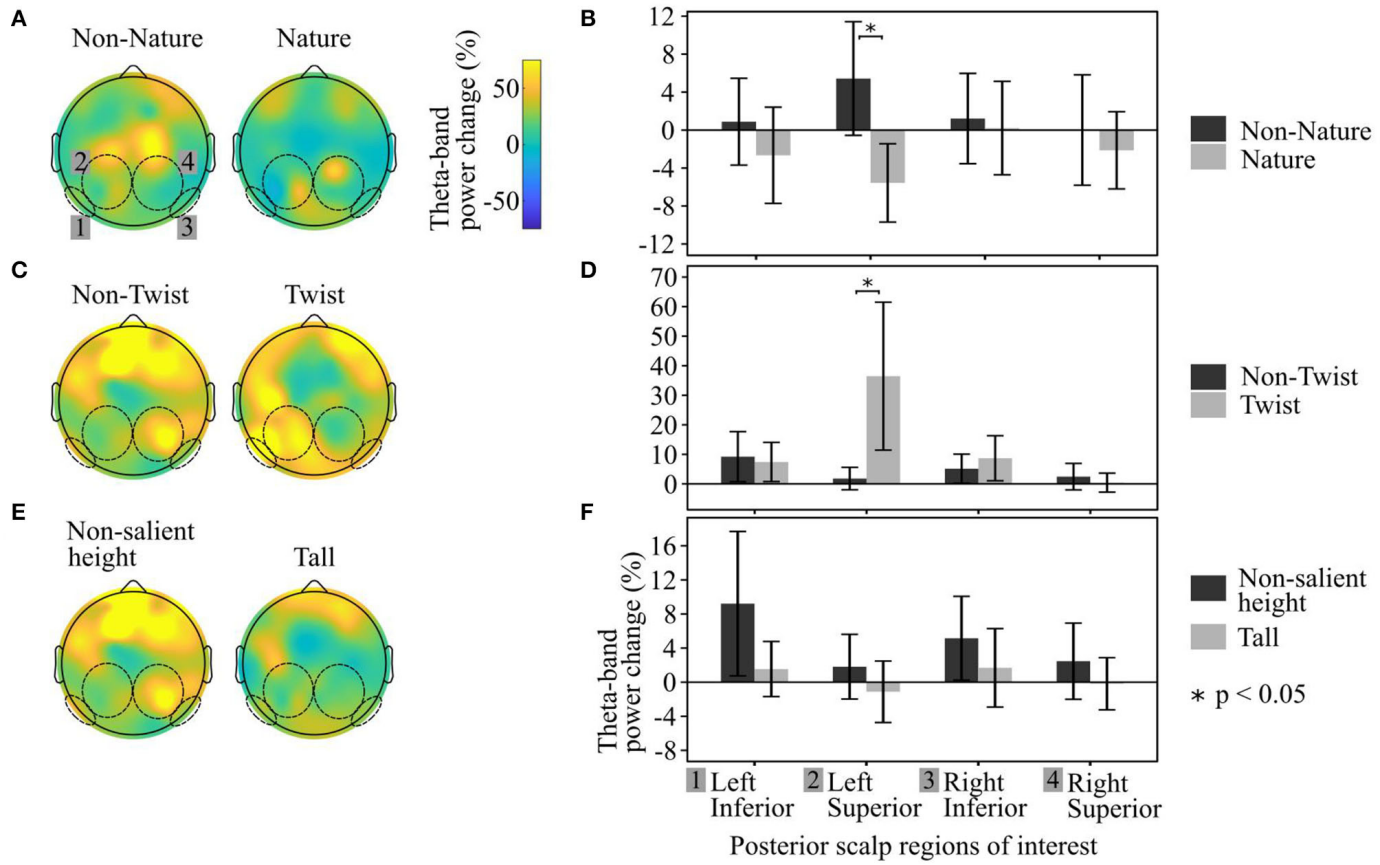
Grand Average Scalp Maps of theta (4.0–7.0 Hz) ERD/ERS when participants observed the buildings inside the VR environment. Comparison for **(A,B)** Non-Nature vs. Nature façade design buildings. **(C–F)** Relative building height. **(A,C,D)** Show the scalp map theta power change compared to baseline. **(B,E,F)** Numerical comparison of theta power change in the four posterior scalp regions of interest to each building condition [in the same left-right order as the scalp-maps in **(A,C,D)**]. Note different y-axis limits.

Individual *t*-tests comparing Twist to Non-Twist façade design buildings within each of the four scalp regions revealed that theta power changes over baseline for the Twist-façade condition were significantly greater than for the Non-Twisted condition in only the Left Posterior Superior region (*t* = 2.08, *p* = 0.01) (Figures 5C,D). The findings in regard to Twisted buildings support Hypothesis H4.

The comparison of the Nature façade design with a corresponding “Non-Nature” building yielded an activation difference in the Left Posterior Superior region (*t* = −2.35, *p* = 0.02). However, the direction of this effect was the opposite of the Twist and overall Saliency comparisons—when viewing the Nature condition (which was recalled more accurately and gazed at longer than most other buildings), participants' Left Posterior Superior regions not only had lower theta power change than the control condition, the Left Posterior Superior theta power was actually *lower* in comparison with the rest/reference window. Conversely, during the Twist façade design and overall Saliency comparisons, the participants' Left Superior Posterior regions had a *higher* theta power over baseline in comparison with their corresponding buildings (Figures 5A,B). Both findings about façades with natural elements and a twist-design façade are salient features that show statistically significant differences in theta power synchronization / desynchronization, and therefore support our proposed hypothesis H3.

The pairwise tests for the relative building-height features did not yield significant associations with the data in regard to any of the EEG regions, although the buildings with the same height as their surrounding were nearly statistically significantly greater in theta power change in the LPI region (*t* = 1.93, *p* >0.06).

Thus, Hypotheses H2 was not supported by the EEG data.

## Discussion and Conclusions

In this study behavioral data, recall accuracy, and EEG data were combined to analyze the effect of architectural features on visual landmark saliency. Our primary Hypothesis H1 was that recall accuracy, user interaction (gaze duration), and neural signatures of spatial awareness in the form of posterior lateral EEG theta power, would be heightened when participants gazed at salient buildings as compared to non-salient buildings. The EEG results support this main effect hypothesis, with the caveat that the progression in posterior theta power change, from less-salient to more-salient buildings, did not seem to act in a linear fashion. Highly salient buildings were also found to attract a greater duration of gazing time on the part of the study participants, and the spatial location of these buildings was more accurately recalled in short-term visual memory tests.

Expanding on our primary hypothesis, we aimed to show that the same behavioral and neural features mentioned above would be heightened when viewing buildings with a specific salient feature, as compared with buildings that lack the corresponding architectural feature. The support for this hypothesis was mixed. EEG results indicated significant differences in neural activation measured at left superior posterior regions of the scalp (Figures 5A–C) for buildings with a Twist-façade design and for buildings in which the façade designs contained natural elements. However, the results for the nature-containing buildings were opposite of what we expected, correlating with a decrease in theta activation (i.e., ERD) at the target scalp area, rather than an increase in theta activation (i.e., ERS). EEG tests for other types of design features did not show significant differences, in some cases diverging from the results found in the gaze duration and recall data. For example, participants gave seemingly more attentional resources to Vertical and Voronoi façade patterns than Horizontal or Grid patterns as evidenced by the gaze and recall results, yet the electrophysiology data did not correspond.

These results support the hypothesis that when people gaze at “interesting” buildings that stand out from the surrounding environment, scalp EEG theta power above posterior parietal cortex increases. Further experiments and analyses, including source-localization steps and higher spatial resolution modalities (e.g., fMRI, MEG), would more conclusively test the retrosplenial cortex's causal role in making some landmarks more effective than others. However, the current experiment provides support and justification for that work. It also suggests that effective landmarks serve as a focus of visual attention, and their location therefore persists more accurately in short-term visual memory. Consistent with the salience model proposed by Raubal and Winter (2002) and the predictive model proposed by Zhang et al. (2014), our findings show that building shape and height can influence the visual attraction of landmarks. However, several open questions remain, which should motivate future work using these and similar methods. For example, what combination of architectural design features are causing this saliency response, and what role does short-term memory play? Do more salient buildings lend themselves to successful use as landmarks simply by virtue of the increased gaze duration, i.e., as a result of spending more time “taking in” that location in the path/environment? Or do these landmarks that stand out visually recruit more attentional resources during perception? Do other regions involved in spatial navigation and memory, such as the hippocampus, show more effective connectivity with the RSC while during memory encoding, or do more salient buildings instead function better as landmarks by boosting simply recall? Or do they lead to improvements in both encoding and recall? These questions can be answered with further experiments building on this research platform.

Due to the fact that in this experiment participants did not engage in ambulatory locomotion (they were seated), and within the virtual environment they did not have the option of truly free navigation (they simply moved among pre-determined locations along the path, i.e., via “teleporting”), we can only make limited generalizations about wayfinding based on these results alone. Nevertheless, this is an important next step in understanding the psychological role of design decisions and architectural features of an environment. While the minimization of movement-related artifacts helps strengthen the quality of combined VR-EEG research, ongoing advances in mobile EEG technology will enable researchers to test the validity of virtual navigation studies. It is also important to note that locomotion may not be as necessary for visual attention as it is for wayfinding, and since this was not a wayfinding study, the absence of locomotion and vestibular input may not a major flaw. Future work would need to test whether free ambulatory locomotion would disproportionately affect visual attention and retrosplenial connectivity with other spatial memory and navigation centers more for distinctive buildings than non-distinctive buildings, perhaps by offering more gradations of perspective and longer, more dynamic periods in which the buildings are partially visible.

Regarding specific architectural features, our finding that the twisted façade was associated with significant activation in the left superior parietal areas of the scalp indicate that some architectural features inspire wayfinding-related activation (Figures 5C,D). This is corroborated by the finding that the twisted buildings tended to become a strong focus of our participants' visual attention (Figure 3), even when the location recall accuracy differed from both buildings that contained this salient feature. Based on an evaluation of recall responses, it seems that participants often confused the two Twist-façade buildings with each other during the recall step, which is the likely cause of the discrepancy between the EEG theta results and the location recall accuracy results.

Our finding that increased recall accuracy and gaze time generally accompanied increases in Left Posterior Superior theta power change for *a-priori-*defined salient buildings, but an opposite pattern for the building with “green” or “nature elements” incorporated, suggests that different kinds of landmark saliency may act on our navigational attention systems and even our default mode network, differently. Further neural research in this domain should investigate connectivity changes in response to different landmarks, as no brain region acts alone.

The findings of this research have implications for urban planners and metropolitan authorities in their goal of developing better wayfinding systems. Previous studies have shown that landmarks are a crucial element in pedestrian and vehicle navigation (Lynch, 1960; May et al., 2003; Goodman et al., 2005; Reagan and Baldwin, 2006; Millonig and Schechtner, 2007; Stark et al., 2007; Hile et al., 2008). However, landmarks are rarely included in route descriptions and other urban wayfinding literature due to the problem of determining what environmental features should be identified and promoted as landmarks (Elias, 2003; Duckham et al., 2010; Peters et al., 2010; Winter et al., 2008). A more robust understanding of what urban features are most useful for diverse participants in landmarking—based on meaningful isolation of design variables and scientific data-collection—has the potential to help solve this dilemma and establish more reliable guidelines for the selection of urban landmarks in wayfinding communications.

## Data Availability Statement

The raw data supporting the conclusions of this article will be made available by the authors, without undue reservation.

## Ethics Statement

The studies involving human participants were reviewed and approved by Institutional Review Board at the University of Houston. The patients/participants provided their written informed consent to participate in this study.

## Author Contributions

SK developed the experimental design and oversaw the collection of data. JC-G collected the data. JC-G, JR, and SK contributed to the behavioral and physiological data analysis, the interpretation of the results, and wrote the manuscript. JR performed statistical analyses. All authors contributed to the article and approved the submitted version.

## Conflict of Interest

The authors declare that the research was conducted in the absence of any commercial or financial relationships that could be construed as a potential conflict of interest.
