# Supplementary material for: Using Posterior EEG Theta Band to Assess the Effects of Architectural Designs on Landmark Recognition in an Urban Setting
Source: Front Hum Neurosci. 2020 Dec 11;14:584385. doi: 10.3389/fnhum.2020.584385 (PMC7759667; doi:10.3389/fnhum.2020.584385)
Supplement: Supplementary file 1 [file Table_1.pdf]

Building Description	Mean Gaze Time (s)	Gaze Time Std. Dev. (s)	Mean Gaze Count	Gaze Count Standard Deviation	Order in route 1	Order in route 2	Mean Location Recall Accuracy
B10<br>Footprint: Rectangle<br>Façade: Twist	11.91	6.94	3.75	2.10	3	9	17.86%
B08<br>Footprint: Rectangle<br>Façade: Nature	9.76	6.84	2.75	1.92	2	12	86.21%
B11<br>Footprint: Rectangle<br>Façade: Voronoi	8.17	4.66	2.57	1.26	8	1	62.07%
B12<br>Footprint: Pentagon<br>Façade: Twist	7.91	6.64	2.46	1.71	7	2	75.00%
B07<br>Footprint: Rectangle<br>Façade: Vertical	7.87	5.71	2.21	1.10	5	8	65.52%
B01<br>Footprint: Rectangle<br>Façade: Horizontal	7.01	5.23	2.50	1.80	6	4	46.43%
B09<br>Height: Tall<br>Footprint: Rectangle<br>Façade: Grid	6.80	5.37	2.89	1.97	—	—	—
B02<br>Footprint: Rectangle<br>Façade: Horizontal w/ overhang	5.28	6.93	2.00	2.23	—	—	—
B06<br>Footprint: Circle<br>Façade: Grid	5.23	4.82	1.93	1.68	1	6	35.71%
B05<br>Footprint: Rectangle<br>Façade: Grid	4.90	4.43	2.29	1.51	10	3	31.03%
B03<br>Footprint: Rectangle<br>Façade: Grid	3.85	4.45	1.54	1.32	12	11	27.59%
B04<br>Footprint: Rectangle<br>Façade: Grid	3.39	2.83	1.43	1.14	11	10	14.29%
